# Multimorbidity in tuberculosis (TB) and its impact on patient care (MITICare): a cross-sectional study nested within a prospective cohort study protocol.

**DOI:** 10.12688/wellcomeopenres.24237.1

**Published:** 2025-05-28

**Authors:** Katherine Hill, Rogers Owori, Molly Naisanga, Noela Owarwo, Sarah Mills, Helen R Stagg, Stellah Mpagama, Christine Sekaggya-Wiltshire, Derek Sloan

**Affiliations:** 1 School of Medicine, Infection and Global Health, University of St Andrews Faculty of Medicine, St Andrews, Scotland, KY16 9AJ, UK; 2 Infectious Diseases Institution, Makerere University Infectious Diseases Institute, Kampala, Central Region, 22418, Uganda; 3 Gulu University, Gulu, Northern Region, Uganda; 4 London School of Hygiene and Tropical Medicine Faculty of Epidemiology and Population Health, London, England, UK; 5 Research Department, Kibong'oto Infectious Diseases Hospital, Sanya Juu, Kilimanjaro Region, Tanzania

**Keywords:** Multimorbidity; Tuberculosis; Uganda; Non-communicable disease; Co-morbidity; Mental health.

## Abstract

Multimorbidity, defined as two or more co-existing long-term health conditions, is increasing in low- and middle-income countries, overlapping with ongoing high tuberculosis (TB) incidence. It is known that there is a high prevalence of multimorbidity in patients with TB in South Africa, but our understanding of how common TB-multimorbidity is in other African countries, and its effect on the trajectories of TB care, is limited. This cross-sectional study nested within a prospective cohort (co-designed between the Infectious Diseases Institute, Uganda and the University of St Andrews, United Kingdom) aims to describe the burden and evaluate the consequences of multimorbidity among patients with TB disease in Kampala, Uganda. The primary objective is to describe the prevalence of multimorbidity at the start of treatment for TB. The secondary objectives are to determine the effect of multimorbidity on clinical characteristics at the start of treatment, progress through TB care, and end of TB treatment outcomes. 254 adults commencing treatment for TB shall be recruited. Multimorbidity shall be assessed using structured questionnaires, simple examination and blood analysis. Th clinical characteristics of TB shall be determined using health and quality of life scores and, in patients with pulmonary TB, the degree of chest X-ray abnormalities and sputum bacillary burden. Patients shall be followed-up at two and six months and their response to treatment determined. The analysis of the prevalence of multimorbidity at baseline shall be reported using a proportion and 95% confidence interval. For the secondary objectives, regression models adjusting for confounders identified through directed acyclic graphs will be used. This study has been developed in close collaboration with a core patient and public involvement group, who will also be actively involved in the dissemination of study results. Ugandan and St Andrews University ethical approval has been prospectively granted (IDI-REC-2023-82, MD17720 and HS3888ES).

## Plain Language Summary

Multimorbidity is the presence of two or more long-term health conditions. Multimorbidity is increasing in Uganda, where there also continues to be high rates of tuberculosis (TB), a bacterial disease mostly associated with the lungs. Multimorbidity may increase the risk of developing and having a poor outcome from TB. The impact of multimorbidity in patients with TB in Uganda is poorly understood. This study, “Multimorbidity in Tuberculosis and Its Impact on Patient Care (MITICare)”, aims to close that knowledge gap. Patients who are starting treatment for TB in Kampala, Uganda, will be recruited into this study. Their physical and mental health will be characterised through questionnaires, examination and simple blood tests. They will then be reviewed after two and six months to monitor their health during treatment and assess outcomes. In patients with TB infecting the lungs, at six months we shall also use simple tests to determine if they have lung damage at the end of TB treatment.

## Introduction

Multimorbidity, defined as two or more co-existing physical or mental health conditions, is a substantial healthcare challenge associated with reduced quality of life and life expectancy ^1– 7^. Economic development, urbanisation, and lifestyle change are all driving an increased burden of non-communicable disease (NCDs) in low- and middle- income countries (LMICs) but the patterns and impacts of multimorbidity in LMICs remain poorly understood ^8– 10^. This epidemiological transition towards increasing NCD prevalence is occurring without a preceding reduction in infectious diseases prevalence. This “perfect storm” is further compounded by the healthcare system which has evolved as composites of single-disease clinics and is poorly equipped to manage patients with multiple interacting health conditions ^8, 10, 11^.

Tuberculosis (TB), a bacterial infection primarily caused by *Mycobacterium tuberculosis* (Mtb), is a leading cause of death worldwide, the major burden of which falls in LMICs ^12^. Shared biological and social determinants drive the overlap between TB and NCDs and may contribute to unsuccessful TB treatment outcomes ^13– 16^. In 2022, the WHO published a framework for collaborative action on TB and co-morbidities, which identified an urgent need to address multimorbidity holistically, rather than as a series of individual TB-co-morbidities ^17^. However, our understanding of the effect of multimorbidity on trajectories of TB care is limited. Epidemiological studies in LMICs have found a high prevalence of multimorbidity in TB patients, which adversely affects treatment outcomes ^18, 19^. However, African studies are largely from South Africa, where the socioeconomic context is different from that of other parts of the continent ^20^ and patterns of multimorbidity may not be comparable. The Infectious Diseases Institute (IDI) is a centre of clinical and research excellence, serving people living with HIV (PLWHIV) in Kampala, Uganda. IDI and associated Kampala Capital City Authority (KCCA) clinics treat 420 new TB patients per year. This cross-sectional study nested within a prospective cohort, co-designed by IDI and the University of St Andrews, aims to describe the burden and evaluate the consequences of multimorbidity among patients with active TB disease in Kampala, to inform policymaking on integrated care.

## Protocol

Adult patients will be recruited within the first 30 days of commencing TB treatment from clinics in or associated with IDI and KCCA. Participant selection will not be restricted by site of TB. Participants with pulmonary TB will be defined as those with Mtb detected on sputum, typical chest X-ray changes or those with a clinical diagnosis of pulmonary TB made by a clinician, on the basis of symptoms. Pulmonary TB participants will undergo additional sputum analysis and review of chest radiological changes as detailed below. Patients will then be followed up for six months from enrolment.

### Objectives and endpoints

The primary objective of this study is:

- To describe the prevalence of multimorbidity in patients at the start of treatment for TB in Kampala. This will be measured through the nested cross-sectional study

The secondary objectives are:

- To determine the effect of multimorbidity on patient health and clinical characteristics of TB at the start of treatment. This will be measured using the following end-points determined in the nested cross-sectional study:
  - Patient health and quality of life scores at baseline, using the Functional Assessment of Chronic Illness Therapy-Tuberculosis (FACIT-TB) and 5Q-5D-5L, in all TB patients
  - Degree of chest X-ray abnormalities in patients with pulmonary TB
  - Sputum bacillary burden in patients with pulmonary TB, measured where available by sputum smear grading, Xpert MTB-RIF and TB-Molecular Bacterial Load Analysis (TB-MBLA).

- To determine the effect of multimorbidity on TB treatment outcomes. This will be assessed using the following endpoints measured in the prospective cohort:
  - 2021 WHO-defined end of TB treatment outcomes: treatment success (composite of cured and completed), treatment failure, death and lost to follow-up ^21^.
  - Patient health and quality of life scores after six months of TB treatment, using 5Q-5D-5L and FACIT-TB.
  - Evidence of post-tuberculosis lung disease (PTLD) after six months of TB treatment using symptom screening, six-minute walking test (6MWT), repeat chest X-ray and/or spirometry in participants with pulmonary TB.

- To determine the effect of multimorbidity on progress through TB care. This will be assessed using the following endpoints measured in the prospective cohort:
  - Number and severity of adverse events during the first six months of TB treatment.
  - Sputum bacillary burden in patients with pulmonary TB at two months of TB treatment (as compared to baseline), using TB-MBLA.
  - Mental health scores at two and six months of TB treatment (as compared to baseline), using the Patient Health Questionnaire-9 (PHQ-9) and the Generalised Anxiety Disorder Questionnaire-7 (GAD-7).
  - Nutritional status at two and six months of TB treatment (as compared to baseline).

Questionnaires and tools used are described in study evaluations and Table 3.

### Participant selection and withdrawal

Participants must meet all of the following inclusion criteria at time of screening to be eligible for enrolment into the study:

1. Evidence of a personally signed and dated informed consent document indicating that the participant (or a legal representative) has been informed of all pertinent aspects of the study.
2. Participants who are willing and able to comply with scheduled visits, treatment plan, laboratory tests, and other study procedures.
3. Aged 18 years or over.
4. Commencing treatment for active TB disease.

Participants presenting with any of the following will not be included in the study:

1. Those already more than 30 days into anti-TB treatment
2. Those already recruited into an interventional treatment trial whose investigator deems that recruitment into this observational study will potentially interfere with interventional trial outcomes.

This study has a core patient and public involvement (PPI) team of three TB survivors based in Kampala who have advised on strategies to recruit and retain patients, and who will help to develop links with the Community Advisory Boards. Through this, awareness of and the benefits to patients of being involved in this study will be publicised. The timings of follow-up assessments have been selected to be co-ordinated with routine TB clinic appointments to minimise participant inconvenience. Many of the follow-up assessments can be performed via telephone, if face-to-face follow-up is not feasible. To describe the TB population as representatively as possible, we shall include pregnant women, people with disabilities and other vulnerable groups that meet the above stated inclusion and exclusion criteria. There is no additional anticipated risk to these patient groups as this is an observational study.

Participants may withdraw from the study at any time at their own request, or they may be withdrawn at any time at the discretion of the investigator or sponsor if the participant meets an exclusion criterion (either newly developed or not previously recognised) that precludes further study participation or is unable to comply with the protocol required schedule of study visits or procedures. The primary outcome, on which the study sample size has been calculated, is assessed at point of enrolment into the study. Therefore, there shall be no replacement of participants who withdraw early. 

### Sample size

The sample size is based on the primary outcome of cross-sectional multimorbidity prevalence assessment. Peltzer *et al.* found a multimorbidity prevalence of 20.9% in new TB and TB retreatment patients in South Africa ^22^. In the absence of data on multimorbidity in TB patients in Uganda, this prevalence was used for a precision calculation in “preszie” R-statistical package with a 95% confidence interval (CI). The Agresti-Coull method for CI generation was used as recommended by Brown *et al.* ^23^. As a pragmatic choice between the precision of the prevalence estimate and feasibility within the project timeframe, a precision of 5% additively applied on either side of the prevalence estimate has been selected which requires a sample size of 254 patients ( Table 1).

**Table 1.** Sample size calculation (precision).

**% Precision**	±1%	±2%	±3%	±4%	±5%	±6%	±7%	±8%	±9%
**Anticipated confidence** **interval (CI)**	19.9% - 21.9%	19.0% - 23.0%	18.1% - 24.1%	17.2% - 25.2%	16.3% - 26.3%	15.5% - 27.5%	14.7% - 28.7%	14.0% - 30.0%	13.3% - 31.3%
**Sample size**	6351	1588	706	397	254	176	130	99	78

### Study visits

**Figure 1.**
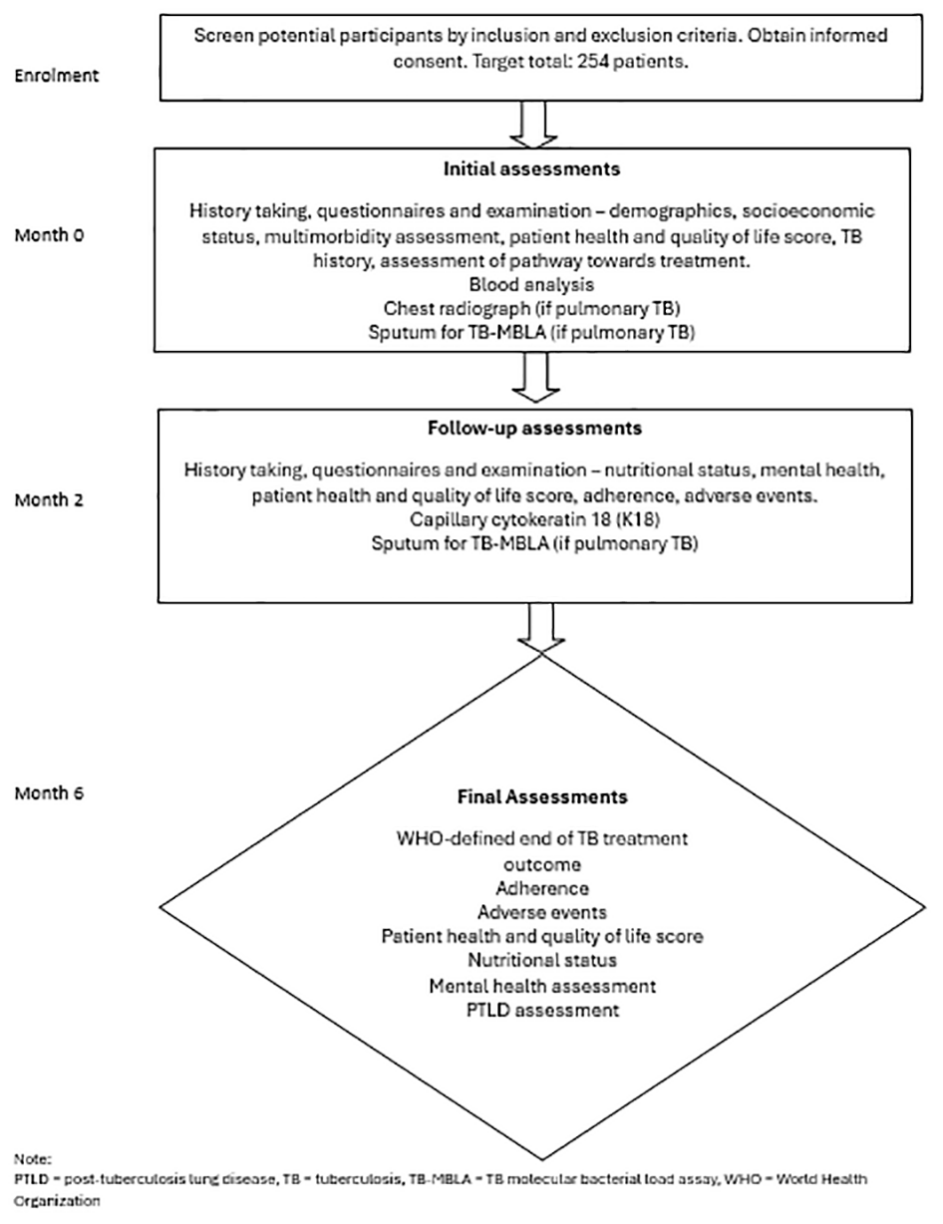
Schematic of study design.

Study visits are summarised in Figure 1 and Table 2.

**Table 2.** Study schedule of events.

Protocol Activity	Screen & Day 0	Month 2	Month 6
Informed Consent	X		
Demographics: age, gender, socio-economic status, educational level, occupation	X		
TB history including site, sensitivity status and any previous episode of TB	X		
Multimorbidity analysis through history taking, WHO STEPS tool and blood analysis	X		
Blood K18	X	X	
Mental health assessment (PHQ-9, GAD-7)	X	X	X
Nutritional assessment	X	X	X
TB-MBLA on sputum (pulmonary TB participants only)	X	X	
Chest X-ray (pulmonary TB participants only)	X		X
TB symptom profile/ Participant reported health and functional status	X	X	X
Adverse events		X	X
Adherence		X	X
WHO TB treatment outcome			X
PTLD assessment			X

Note: GAD-7 = generalized anxiety disorder questionnaire, K18 = cytokeratin 18, PHQ-9 = patient health questionnaire-9, PTLD = post-tuberculosis lung disease, TB = tuberculosis, TB-MBLA = TB molecular bacterial load assay, WHO STEPS = World Health Organization STEPwise approach to NCD risk factor surveillance.

***Screening***

Eligible patients will be informed about study procedures by a member of the research team and given written patient information that is available in English and/or Luganda. Consent procedures will occur once the patient has had sufficient time to contemplate their decision. A follow-up meeting will be arranged if there is insufficient time on the first day of contact. Signed informed consent will be obtained from all patients that meet the eligibility criteria and agree to participate in the study. The consent form will be stored securely in a locked cabinet in the study office. A copy of the consent form shall be given to each participant to keep. Once the consent form has been completed, the participant will be assigned a unique study identification number.

The following procedures shall be performed at study visits.

***Enrolment visit (Day 0 – 30)***

- Schedule study visits for the duration of the study.
- Provide participants with information on the study aim and follow-up schedule.
- Obtain and record demographic and socioeconomic information.
- Obtain and document any known medical history and medications.
- Review and obtain copies of the results of any relevant laboratory tests (including HIV Ag/Ab, CD4 count, HIV viral load, hepatitis B surface antigen) from reporting laboratories.
- Undertake a multimorbidity, nutritional and mental health assessment through history taking, structured questionnaires and examination.
- Collect blood samples for analysis as detailed below.
- All participants should already be screened for HIV, but if not then an HIV status test shall be undertaken as part of this study.
- Obtain details of TB diagnosis including duration of symptoms, date of diagnosis, evidence for diagnosis, site and drug susceptibility profile, if known.

- An assessment of TB symptom profile and participant reported health and functional status using:
  - FACIT-TB.
  - 5Q-5D-5L.

- For pulmonary TB patients only, the following additional tests will be done:
  - Take or review chest radiograph.
  - Sputum for TB-MBLA may also be collected.

***Month two visit (Day 60 ± 7)***

- Record adverse events as reported by participants or observed by the investigators, quantified using Common Terminology Criteria of Adverse Events (CTCAE) for grading of adverse event severity.
- Record participant self-reported adherence.
- Record assessment of TB symptom severity and participant reported health and functional status using 5Q-5D-5L and FACIT-TB.
- Record nutritional state – height, weight, waist and mid-upper arm circumference, and FAO food insecurity questionnaire.
- Assess mental health using Patient Health Questionnaire (PHQ-9) and Generalised Anxiety Disorder Assessment (GAD-7).
- Capillary K18 may be done.
- For pulmonary TB patients only, sputum for TB-MBLA may be collected.

***Month six visit (Day 180 ± 7)***

- Record adverse events as reported by the participant or observed by the investigators, quantified using common toxicity criteria for grading of adverse event severity.
- Record participant self-reported adherence.
- Record assessment of TB symptom severity and participant reported health and functional status using 5Q-5D-5L and FACIT-TB.
- Record nutritional state – height, weight, waist and mid-upper arm circumference, and FAO food insecurity questionnaire.
- Assess mental health through PHQ-9 and GAD-7.
- Record WHO-defined end of TB treatment outcome for those who have completed treatment course at 6 months.
- For pulmonary TB patients only, repeat chest radiograph, 6MWT and spirometry may be performed.

For participants who have not completed TB therapy at 6 months, the WHO-defined end of TB treatment outcomes shall be collected from TB registry documents at the relevant time without further contact with the study participant.

***Missed visits***

Participants who do not attend the month two or month six visit shall be called by the study team and an alternative appointment within the specified time period will be arranged. If it is not possible to arrange face-to-face assessment, data that can be collected via telephone shall be obtained. If it is not possible to contact participant by telephone, information from medical records shall be obtained where possible.

***Withdrawal visit***

If the participant withdraws early or the investigator terminates participant participation, end of TB treatment outcome will be obtained through use of TB registry documentation and no other additional data will be acquired.

***Unscheduled visit***

Participants will be given contact numbers for the study team, in case they have questions or concerns at any time. Unscheduled visits may be arranged as required. If additional medical needs are identified, participants will be referred to the appropriate clinical service.

### Study evaluations

***Questionnaires***

Several quantitative structured questionnaires have been incorporated into this study, which are described in Table 3. For patients with pulmonary TB, 6MWT and spirometry may also be performed at month 6 to assess post-TB lung function. The 6MWT is a simple and easy to perform measure of lung functional capacity. Patients are asked to walk continuously for 6 minutes around a track of defined distance and the total distance covered at the end of 6 minutes is recorded ^24^.

**Table 3.** Study Evaluations.

**World Health Organization (WHO) ** **STEPwise approach to NCD risk** ** factor surveillance (STEPS)**	The WHO STEPS has been developed to allow standardised methods of diagnosing and reporting non-communicable diseases (NCDs) across countries. It is a composite of questionnaires, simple physical measurements and collection of blood samples for limited biochemical analysis and is designed to detect the key NCDs in a standardized and practical approach. We shall be using this tool and the tests included in the section below.
**5Q-5D-5L**	The 5Q-5D-5L is a health-related quality of life assessment tool that has been validated across a broad range of populations, settings and conditions and has been used extensively in research ^26^. We shall be using this to capture quality of life in this patient group.
**Functional Assessment of Chronic** **Illness Therapy-Tuberculosis** ** (FACIT-TB)**	WHO TB treatment outcomes, which focus on either microbiological cure or completion of prescribed treatment, do not capture the physical, mental, and psychosocial health impairments that TB causes and which may remain impaired even after microbiological cure ^27^. Disease-specific quality of life tools are needed to capture these. FACIT-TB has been developed for this purpose ^28^ and we shall be using it as a patient reported outcome measure in this study.
**Karnofsky performance status**	Karnofsky performance score is a simple patient functional assessment tool ^29^. It is used routinely within clinics at the Infectious Diseases Institute (IDI) and so will be comparable to local data and clinical practice.
**Patient Health Questionnaire-9 ** **(PHQ-9)**	The PHQ-9 is a depression assessment tool, comprising of 9 self-rated questions, that has been translated into several Ugandan languages. It has been validated for use in Uganda, with a sensitivity and specificity of 92% and 89% respectively in the HIV population and is in routine use in IDI ^30^.
**Generalised Anxiety Disorder ** **Questionnaire (GAD-7)**	The GAD-7 is an anxiety assessment tool, comprising of 7 self-rated questions, that has been validated across several Sub-Saharan African countries ^31^.
**Food and Agriculture Organization ** **of the United Nations Food** ** Insecurity Experience Scale (FIES)**	The FIES is a metric of food insecurity severity and has been designed to be comparable across different countries and sub-national populations ^32^. This is being included within the nutritional assessment of participants to help delineate whether malnutrition is attributable to poor physical health or food insecurity.

***Blood analysis***

The following analyses will be performed on the participant blood samples on the day of enrolment into the study:

- Full blood count (if not done within last 7 days),
- Creatinine,
- Alanine transferase (ALT), in addition K18 may be done, which has previously been shown to detect liver toxicity earlier than ALT ^25^,
- Bilirubin (total),
- Haemoglobin A1c (HbA1c) (if not done within the last 7 days),
- HIV testing (if not already known),
- CD4 count (in PLWHIV and if not done within preceding 3 months). The most recent HIV viral load result will be obtained, where possible, from reporting laboratory,
- Hepatitis B surface antigen (HBsAg) (if not already known).

The total volume of blood obtained by venepuncture shall be approximately 20ml. All blood samples shall be processed in the IDI Core Laboratory using locally agreed standard operating procedures.

***Sputum analysis***

In participants with pulmonary TB, copies of the results of mycobacterial sputum analysis already performed within 14 days of commencing TB treatment shall be obtained from the reporting laboratories, including smear grading, cycle threshold of Xpert MTB/RIF, time to culture positivity and TB-MBLA, where available. TB-MBLA, which has been validated to determine viable mycobacterial load, may be conducted at baseline and month two to assess treatment response ^33^. The inclusion of this analysis will be dependent on additional funding.

***Radiography***

A chest X-ray will be performed - or a radiograph reviewed wherever possible if already done -, in all participants with pulmonary TB within 30 days of commencing TB treatment. The result of the chest radiograph will be documented and a photograph of the radiograph obtained. A repeat chest radiograph at month 6 may also be performed, to assess for PTLD.

### Patient and Public Involvement (PPI)

A core PPI team of three Ugandan TB survivors has been regularly consulted during the design of this study. Specifically, they have shaped the overall study design, ensured feasibility and acceptability of all study evaluations and advised on perceptions on study variables that may carry stigma (e.g. mental health and substance use). All patient facing material including informed consent forms have been co-developed to ensure clear and accurate communication. They have advised on strategies to recruit and retain patients, and will help to develop links with the Community Advisory Boards. Through this, awareness of and the benefits to patients of being involved in this study will be publicised. Additionally, a panel of eight Ugandan TB survivors has been formed and consulted to validate study acceptability and raise study awareness. This PPI input is essential to the study’s success, and both the core and panel PPI groups will continue to be actively involved in delivery of the study and dissemination of key findings.

### Safety considerations

This is an observational, non-interventional cohort study acquiring samples and data only. Risks associated directly with participating are minimal. Any risk associated with sampling is mitigated by ensuring all research samples are collected following local procedures by appropriately qualified and trained members of the clinical and research team. Through the detailed assessment of patients’ physical and mental health status, it is possible that medical conditions will be identified that were not previously known. Diagnosis and management of newly identified health conditions will be managed according to local procedures and referral systems. Participants’ mental health shall be assessed using PHQ-9 and GAD-7 scores, as described above. If a patient scores greater than or equal to 10 on the PHQ-9 in keeping with major depression, or greater than or equal to 10 on the GAD-7 in keeping with moderate to severe anxiety, and this is a diagnosis that their responsible clinician was not aware of, they shall be referred to the psychiatry team at IDI or Mulago Hospital. If suicidal thoughts are expressed at any frequency, this referral shall be arranged for the same day and patient shall remain under the direct supervision of the clinic until this occurs.

### Data analysis and statistical plan

All analyses will be done in R.

***Primary endpoint***

The proportion of patients with multimorbidity at baseline will be calculated with a 95% CI. We are also intending to present prevalence estimates and CIs for different strata of the following variables: age, sex and socioeconomic status. 

***Secondary endpoints***

The secondary endpoint of WHO TB treatment outcomes shall be grouped into the binary categories of “successful” (composite of “cured” or “completed treatment”) or “not successful” (composite of all other outcomes). Logistic regression will be used to examine the association between multimorbidity and TB treatment outcome. For the other secondary endpoints, where possible the following regression models shall be performed:

- Multinomial regression will be used to examine the association between multimorbidity and degree of chest X-ray changes at start of treatment.
- Linear regression with a transformed outcome will examine the association between multimorbidity and quantified bacillary burden at month 0 and month 2. Other regression techniques will be used if data remain skewed.
- A Poisson regression analysis shall be done looking at association between multimorbidity and the rate of adverse events.
- Linear regression shall be done for all other endpoints.

After producing unadjusted estimates, we will then adjust as appropriate for confounding. Directed acyclic graphs (DAGs) have been used to identify the minimal confounder sets that would be used to adjust the regression models. Colinear variables will be detected during the analysis process and handled as appropriate. Potential effect modifiers have also been identified and the results of the regression models will be stratified according to these variables. Missing data will be handled as per the guidance of Lee *et al.* ^34^.

## Ethics and consent

Ethical approval for this study was prospectively sought and approved by IDI Research Ethics Committee (on 23 ^rd^ February 2024, IDI-REC-2023-82), the University of St Andrews School of Medicine Ethics Committee (on 4 ^th^ April 2024, MD17720) and the Uganda National Council of Science and Technology (UNCST) (on 7 ^th^ May 2024, HS3888ES). The study will be conducted in accordance with the protocol, GCP guidelines, legal and regulatory requirements and the general principles set forth in the International Ethical Guidelines for Biomedical Research Involving Human participants and the Declaration of Helsinki. Written informed consent shall be obtained from all participants, or their legal representative, before any study-specific activity is performed. No identifiable data will be shared or published. Data shall be held on secure IDI or University of St Andrews servers. 

## Study dissemination

The core PPI group will be actively engaged in identifying ways to disseminate information before, during and after the study to relevant populations. We shall co-produce lay interpretations of all study outputs, that will be disseminated via visual summaries for TB clinics, traditional and social media outlets, and community events. The findings will be shared in peer-reviewed, open-access, journals. In addition, the results will be presented at relevant national and international conferences, and to key stakeholders in Uganda.

## Data Availability

Data generated will be held in pseudo-anonymised form to allow analysis and completion of the associated papers. Once these are complete, patient data will be returned to IDI as data controllers. Future access to the data will be on application to the clinics in line with their existing governance policies. All code will be made publicly available via GitHub.

## Data availability statement

No data are associated with this article.
