# Supplementary material for: Multimorbidity in tuberculosis (TB) and its impact on patient care (MITICare): a cross-sectional study nested within a prospective cohort study protocol
Source: Wellcome Open Res. 2025 May 28;10:282. [Version 1] doi: 10.12688/wellcomeopenres.24237.1 (PMC12335725; doi:10.12688/wellcomeopenres.24237.1)
Supplement: Peer review history

# Reviewer report: Reviewer response for version 1

**DOI:** 10.21956/wellcomeopenres.26732.r126516

**Published:** 2025-08-25

**Authors:** Jonathan E Golub

**Affiliations:** 1 Johns Hopkins University, Baltimore, Maryland, USA

This is an interesting study looking at a topic of strong interest in recent years – the prevalence and impact of comorbidities diagnosed at the time of TB diagnosis. These data are needed to develop screening and management programs for people with TB. Many such analyses are ongoing, but this will be one of the first in Uganda. The protocol is clearly described but there are some issues that can be clarified.

 It would be ideal to recruit patients within a shorter window if possible. When a person with TB starts TB treatment, they start to feel better within a few days, and if drug susceptible, certainly within a couple of weeks. Thus, asking about QOL and functional status may be different at time of diagnosis then at 30 days past. Blood glucose levels could start to return to normal and people who quit smoking due to symptoms may have restarted (which is actually a plus for asking later).

 Along these lines, is it possible to test for diabetes at end of TB treatment? Transient diabetes has been well described for people with TB, with blood glucose elevated at time of diagnosis, but reduced/normalized at end of TB treatment. Measuring tobacco use, alcohol use, mental health, etc would be nice at end of treatment as well. Several outcomes are being assessed at month 6 but these are not specifically mentioned.

 Exclusion criteria – interesting that anyone already recruited into an interventional treatment trial will NOT be included in this study, if the investigator of the OTHER trial deems that recruitment into this observational study will potentially interfere with interventional trial outcomes. I would think that they would be worried about the intervention study interfering with the secondary objectives of this study.

 Inclusion of TB survivors to help develop strategies to co-develop consent forms, recruit and retain patients and help work with the CAB is great to see.

 The authors state that “Potential effect modifiers have also been identified and the results of the regression models will be stratified according to these variables.” Can they provide these effect modifiers? It is difficult to understand what confounders, effect modifiers there are when so many variables are being considered, or need to be considered in an analysis of TB treatment outcomes.

 Along these lines, are all morbidities treated equally? Is any one morbidity at baseline considered to be multimorbidity? As stated above, this would be concerning if, for instance diabetes at TB diagnosis is considered a comorbidity but it were to change during follow-up unbeknownst to the investigators. And, for secondary analyses, will they account for people with one comorbidity versus several? Are some comorbidities hypothesized to increase poor TB treatment outcomes more than others?

Is the study design appropriate for the research question?

Partly

Is the rationale for, and objectives of, the study clearly described?

Yes

Are sufficient details of the methods provided to allow replication by others?

Yes

Are the datasets clearly presented in a useable and accessible format?

Not applicable

Reviewer Expertise:

Tuberculosis epidemiology is my area of expertise.

I confirm that I have read this submission and believe that I have an appropriate level of expertise to confirm that it is of an acceptable scientific standard, however I have significant reservations, as outlined above.

# Reviewer report: Reviewer response for version 1

**DOI:** 10.21956/wellcomeopenres.26732.r125923

**Published:** 2025-08-23

**Authors:** Ravindra Nath

**Affiliations:** 1 Teerthanker Mahaveer University, Uttar Pradesh, India

Reviewer Query :

1. Since the proposed study involves follow-up at both 2 and 6 months, it may not be accurately described as a cross-sectional design. The authors may wish to reconsider and clearly specify the appropriate study design to avoid confusion during implementation and reporting.
2. In the abstract, it is mentioned that “the analysis of prevalence of multimorbidity at baseline” will be done. It would be helpful if the authors could clarify what analytical methods they plan to use to assess the prevalence of morbidities.
3. References 18 and 19 are cited to describe the impact of co-morbidities on TB outcomes in LMICs. However, these references currently do not include data from countries in the Southeast Asian Region, such as India or Pakistan. Including literature from these regions—if available—could strengthen the contextual relevance of the background and rationale.
4. The authors are advised to specify the expected number of participants who may be excluded due to prior enrollment in other studies. Including this information in the protocol will improve transparency in the proposed recruitment process.
5. For the sample size calculation, in the absence of local data from Uganda, it may be more appropriate to use a conservative estimate (p = 50%) to avoid underestimating the required sample. The current reliance on data from South Africa may result in a smaller sample size, and this should be justified clearly in the protocol.
6. The protocol would benefit from a brief description of the sampling technique planned for participant selection—whether random, consecutive, or convenience sampling. This will help assess the representativeness and generalizability of future findings.
7. Kindly ensure that the ethical approval for this specific study is secured and mention the name of the approving ethics committee in the protocol. This will be essential before study initiation.

Is the study design appropriate for the research question?

No

Is the rationale for, and objectives of, the study clearly described?

Partly

Are sufficient details of the methods provided to allow replication by others?

Yes

Are the datasets clearly presented in a useable and accessible format?

Yes

Reviewer Expertise:

Tuberculosis, Operational Research, Research Methodology

I confirm that I have read this submission and believe that I have an appropriate level of expertise to confirm that it is of an acceptable scientific standard, however I have significant reservations, as outlined above.

# Reviewer report: Reviewer response for version 1

**DOI:** 10.21956/wellcomeopenres.26732.r126519

**Published:** 2025-08-23

**Authors:** Bayodé Roméo ADEGBITE

**Affiliations:** 1 University of Amsterdam, Amsterdam, Netherlands Antilles

This is a good topic. Few studies have been done on TB and comorbidities. My main concern is about the study design

1. The author stated  that the main  design is cross sectional nested with a cohort .I would suggest to the authors to change this to a cohort  design and adjusted the sample size accordingly .The most of objective and endpoint need a follow up , the cohort design is the best or if the cross sectional design is maintained ,the objectives and endpoints should be revised.

2-The sample size calculation should take into consideration a cohort design

 3-If the authors want  to combine the two designs , they should clearly clarify the sample size for each design and the criteria to be eligible for the cohort component

Is the study design appropriate for the research question?

No

Is the rationale for, and objectives of, the study clearly described?

Yes

Are sufficient details of the methods provided to allow replication by others?

No

Are the datasets clearly presented in a useable and accessible format?

Yes

Reviewer Expertise:

Public health -infectious diseaes

I confirm that I have read this submission and believe that I have an appropriate level of expertise to confirm that it is of an acceptable scientific standard, however I have significant reservations, as outlined above.

# Reviewer report: Reviewer response for version 1

**DOI:** 10.21956/wellcomeopenres.26732.r126521

**Published:** 2025-08-19

**Authors:** Saima Afaq

**Affiliations:** 1 University of York, York, England, UK

The protocol “Multimorbidity in Tuberculosis (TB) and Its Impact on Patient Care (MITICare)” presents a well‐argued rationale and clearly stated objectives. Drawing on WHO frameworks and regional studies, the authors convincingly explain why rising non‐communicable disease burdens alongside persistent TB in Uganda warrant investigation, and they explicitly list primary and secondary aims in the “Objectives and Endpoints” section. The study design logically aligns with these aims, enabling both estimation of multimorbidity at treatment initiation and evaluation of its influence on clinical outcomes, as detailed in their schematic and visit schedule.

 Although inclusion and exclusion criteria, sample size justification using the Agresti–Coull method, validated measurement tools (WHO STEPS, FACIT‐TB, PHQ-9, GAD-7, etc.), and a comprehensive analysis plan (regression modeling and missing‐data methods) are described in sufficient detail to guide replication, key methodological components remain under‐specified. Specifically, the protocol does not enumerate which conditions constitute “multimorbidity” or their diagnostic thresholds (for example, precise blood-pressure or glucose cutoffs), and it refers to “locally agreed SOPs” without appending or linking to those documents. In the absence of a data dictionary or mock dataset illustrating variable names, types, and coding conventions, external investigators cannot fully reproduce the data-collection, management, or analysis procedures. Addressing these points will ensure that other researchers can reliably replicate and build upon this important study of TB and co-existing chronic diseases in Uganda.

Is the study design appropriate for the research question?

Yes

Is the rationale for, and objectives of, the study clearly described?

Yes

Are sufficient details of the methods provided to allow replication by others?

Partly

Are the datasets clearly presented in a useable and accessible format?

Partly

Reviewer Expertise:

TB multimorbidity

I confirm that I have read this submission and believe that I have an appropriate level of expertise to confirm that it is of an acceptable scientific standard, however I have significant reservations, as outlined above.

# Reviewer report: Reviewer response for version 1

**DOI:** 10.21956/wellcomeopenres.26732.r126517

**Published:** 2025-08-09

**Authors:** Arohi Chauhan

**Affiliations:** 1 South Asian Institute of Health Promotion, New Delhi, India

This is a well-designed and timely study protocol with a clear rationale and adequate methodological transparency. Once executed, it has strong potential to contribute to global knowledge and inform policy on integrated care for TB and NCDs in LMICs. However, the following things need to be consider:

 Must Address (for scientific soundness):

1. Clarify data sharing conditions more explicitly – though described, a clear statement of when and how others can apply for data access would be helpful.
2. Consider standardizing terminology – e.g., consistently use either "5Q-5D-5L" or "EQ-5D-5L" across the manuscript for clarity.

Suggestions to Strengthen Manuscript:

- Highlight any potential limitations in generalizability (e.g., urban Kampala setting may not reflect rural regions).
- Briefly discuss anticipated challenges such as participant follow-up or resource constraints for lung function testing.

Is the study design appropriate for the research question?

Yes

Is the rationale for, and objectives of, the study clearly described?

Yes

Are sufficient details of the methods provided to allow replication by others?

Yes

Are the datasets clearly presented in a useable and accessible format?

Not applicable

Reviewer Expertise:

Tuberculosis multimorbidity and health literacy

I confirm that I have read this submission and believe that I have an appropriate level of expertise to confirm that it is of an acceptable scientific standard.

# Reviewer report: Reviewer response for version 1

**DOI:** 10.21956/wellcomeopenres.26732.r125918

**Published:** 2025-08-09

**Authors:** Miftahul Falah

**Affiliations:** 1 Universitas Muhammadiyah Tasikmalaya, West Java, Indonesia

Article Title:

 Multimorbidity in tuberculosis (TB) and its impact on patient care (MITICare): a cross-sectional study nested within a prospective cohort study protocol

 1. Is the rationale for, and objectives of, the study clearly described?

 Yes – clearly described

1. The rationale is strong, grounded in the increasing burden of multimorbidity in low- and middle-income countries (LMICs) and the urgent need to understand its implications in TB care.
2. The background provides good references to epidemiological transitions, WHO guidelines, and gaps in existing African data (mainly from South Africa).
3. The objectives are well-articulated:
  1. Primary: To estimate the prevalence of multimorbidity in TB patients at treatment start.
  2. Secondary: To assess its clinical, mental health, and treatment outcomes.

Strengths:

1. Contextual relevance to Uganda
2. Good linkage to WHO's 2022 framework on TB-comorbidity integration

2. Is the study design appropriate for the research question?

 Yes – appropriate and justified

1. The design is a nested cross-sectional study within a cohort, which is methodologically robust for the stated objectives.
2. The cohort structure allows for both baseline analysis (cross-sectional prevalence) and longitudinal outcomes (over 6 months).
3. Justification for sample size is transparent (based on South African prevalence data).

Strengths:

1. Pragmatic, real-world data collection
2. Observational design matches aim to observe impact, not test interventions

3. Are sufficient details of the methods provided to allow replication by others?

 Yes – very comprehensive

1. The protocol includes clear descriptions of:

1. Eligibility criteria
2. Visit schedule (screening, enrolment, months 2 and 6)
3. Clinical and laboratory assessments
4. Use of validated tools (e.g., PHQ-9, GAD-7, FACIT-TB, 6MWT, TB-MBLA)
5. Blood and sputum analysis protocols
6. Statistical analysis plan, including regression models and DAGs

1. Data management and ethical approvals are well addressed.

Minor Suggestion:

 While methods are thorough, data collection tools (e.g., questionnaires) could be included as appendices or provided as links.

 4. Are the datasets clearly presented in a useable and accessible format?

 No – data not yet available

1. The manuscript is a protocol, so no datasets are yet produced.
2. The authors note that data will be made available upon study completion and that code will be shared on GitHub.
3. The data availability statement confirms that future access will follow institutional policies.

Critical Point to Address:

1. Authors should clarify where and how data will be accessed, including:
  1. URL for GitHub repository
  2. Mechanism for requesting data from IDI

Recommendations for Improvement

 Data availability: Provide a placeholder GitHub link or outline application steps to IDI

 Methods: Attach questionnaires as appendix or supplementary files

 Sample size justification: Consider adding sensitivity analysis for lower or higher prevalence rates

Is the study design appropriate for the research question?

Yes

Is the rationale for, and objectives of, the study clearly described?

Yes

Are sufficient details of the methods provided to allow replication by others?

Yes

Are the datasets clearly presented in a useable and accessible format?

No

Reviewer Expertise:

Article Title:Multimorbidity in tuberculosis (TB) and its impact on patient care (MITICare): a cross-sectional study nested within a prospective cohort study protocol Authors: Katherine Hill et al. Journal: Wellcome Open Research (2025) DOI: 10.12688/wellcomeopenres.24237.1 1. Is the rationale for, and objectives of, the study clearly described?Yes – clearly describedThe rationale is strong, grounded in the increasing burden of multimorbidity in low- and middle-income countries (LMICs) and the urgent need to understand its implications in TB care.The background provides good references to epidemiological transitions, WHO guidelines, and gaps in existing African data (mainly from South Africa).The objectives are well-articulated:1)    Primary: To estimate the prevalence of multimorbidity in TB patients at treatment start.2)    Secondary: To assess its clinical, mental health, and treatment outcomes. Strengths:a.       Contextual relevance to UgandaGood linkage to WHO's 2022 framework on TB-comorbidity integration 2. Is the study design appropriate for the research question?Yes – appropriate and justifieda.       The design is a nested cross-sectional study within a cohort, which is methodologically robust for the stated objectives.The cohort structure allows for both baseline analysis (cross-sectional prevalence) and longitudinal outcomes (over 6 months).Justification for sample size is transparent (based on South African prevalence data). Strengths:a.       Pragmatic, real-world data collectionObservational design matches aim to observe impact, not test interventions 3. Are sufficient details of the methods provided to allow replication by others?Yes – very comprehensivea.       The protocol includes clear descriptions of:1)    Eligibility criteria2)    Visit schedule (screening, enrolment, months 2 and 6)3)    Clinical and laboratory assessments4)    Use of validated tools (e.g., PHQ-9, GAD-7, FACIT-TB, 6MWT, TB-MBLA)5)    Blood and sputum analysis protocols6)    Statistical analysis plan, including regression models and DAGsData management and ethical approvals are well addressed. Minor Suggestion:While methods are thorough, data collection tools (e.g., questionnaires) could be included as appendices or provided as links. 4. Are the datasets clearly presented in a useable and accessible format?No – data not yet availableThe manuscript is a protocol, so no datasets are yet produced.The authors note that data will be made available upon study completion and that code will be shared on GitHub.The data availability statement confirms that future access will follow institutional policies. Critical Point to Address:Authors should clarify where and how data will be accessed, including:1.     URL for GitHub repository2.     Mechanism for requesting data from IDI  Recommendations for Improvement Data availability: Provide a placeholder GitHub link or outline application steps to IDIMethods: Attach questionnaires as appendix or supplementary filesSample size justification: Consider adding sensitivity analysis for lower or higher prevalence rates

I confirm that I have read this submission and believe that I have an appropriate level of expertise to confirm that it is of an acceptable scientific standard.
